# Prioritising Sexual and Reproductive Health on a Female Inpatient Psychiatric Ward: A Quality Improvement Project

**DOI:** 10.1192/bjo.2025.10460

**Published:** 2025-06-20

**Authors:** Eleanor Mitham, Katherine Wickham, Elizabeth Pillai, Stella Rosson, Miranda Holliday

**Affiliations:** 1 Homerton University Hospital, London, United Kingdom; 2 North East London NHS Foundation Trust, London, United Kingdom; 3 East London NHS Foundation Trust, London, United Kingdom

## Abstract

**Aims:** Serious mental illness is associated with higher rates of sexual assault and gynaecological cancers, alongside pre-menstrual disorders and the menopause which can be implicated in psychiatric presentation, relapse or treatment resistance. This project aimed to ensure better screening and referral for sexual and reproductive health issues in order to improve relevant health outcomes.

**Methods:** Two pre-existing health-recording forms were highlighted to nursing staff and encouraged using information posters placed in staff areas. Six individual referral pathways were developed for doctors’ use. Data was collected pre- and post-intervention including proportion of forms completed, abnormalities identified, appropriate follow-up initiated, and time spent carrying out these tasks. Reasons for non-completion were analysed. PDSA cycles were used to guide improvements and increase engagement.

**Results:** Prior to intervention, 53% of patients had the ‘women’s physical health’ (WPH) form completed, 0% the contraception form. Despite 59% of these finding abnormalities, 0% were referred for investigation or treatment (32 patients over a 2-month period August–September 2024). Post-intervention, completion of the forms remained static at 50% of WPH and 0% of contraception forms, though detected abnormalities rose to 88% and appropriate referrals to 40%. Of the remaining 60%, 7 patients identified as requiring a referral declined, most commonly refusing a smear test. 1 further patient was too unwell to engage. Overall patient group size was similar with 34 patients over a 2-month period November 2024–January 2025. Average time for form-reviewing and referring was 6.9 minutes per patient. Independently of the forms, 1 patient who remained admitted throughout both data periods was followed up for 3 separate issues, and 4 patients without completed forms were noted to have concerns, and subsequently referred appropriately.

**Conclusion:** Though patient referrals increased from 0 to 40% after the referrals guide was created, the proportion of concerns addressed remained low. Patient education is a key target for improvement, specifically cervical screening, and eye-catching patient education posters are to be displayed on the ward for this purpose. Form-completion rates did not improve, suggesting further engagement with nursing colleagues and specific time-allocation for completion would be of benefit. Perhaps most significantly, the increased identification of reproductive and sexual health concerns of patients without a completed form highlighted the team’s increased awareness of these issues. This suggests that clinician education can help in utilising inpatient admission as an opportunity to improve sexual and reproductive health for women with serious mental illness.

